# Lipid Droplets Accumulation during Hepatitis C Virus Infection in Cell-Culture Varies among Genotype 1–3 Strains and Does Not Correlate with Virus Replication

**DOI:** 10.3390/v13030389

**Published:** 2021-02-28

**Authors:** Andrea Galli, Santseharay Ramirez, Jens Bukh

**Affiliations:** 1 Copenhagen Hepatitis C Program (CO-HEP), Department of Infectious Diseases, Hvidovre Hospital, 2650 Hvidovre, Denmark; andrea.galli@regionh.dk (A.G.); santseharayra@sund.ku.dk (S.R.); 2 Department of Immunology and Microbiology, Faculty of Health and Medical Sciences, University of Copenhagen, 2200 Copenhagen, Denmark

**Keywords:** HCV, hepatitis, cell-culture, genotype, lipid droplet, core, assembly, microscopy

## Abstract

Liver steatosis is a common complication of chronic hepatitis C virus (HCV) infection, which can result in accelerated liver fibrosis development, especially in patients infected with genotype 3a. The precise mechanisms of HCV-induced liver steatosis remain unclear, but it is often posited that increased intracellular lipid accumulation is the underlying cause of steatosis. To study experimentally how HCV infection in human liver derived cells by different genotypes and subtypes might affect lipid accumulation, we performed detailed cytofluorimetric and microscopy analyses of intracellular lipid droplets (LDs) in relation to the viral Core and to cell endoplasmic reticulum proteins. Following culture infection with HCV genotype 1a, 2a, 2b, 2c, and 3a strains, we found variable levels of intracellular LDs accumulation, associated to the infecting strain rather than to the specific genotype. Although two genotype 3a strains showed high levels of lipid accumulation, as previously observed, some strains of other genotypes displayed a similar phenotype. Moreover, the analyses of LDs size, number, and shape indicated that the apparent increase in lipid accumulation is due to an increase in the overall number rather than in the size of droplets. Finally, differences in total lipid content across genotypes did not correlate to differences in Core distribution nor Core levels. In conclusion, our study provides a quantitative in-depth analysis of the effect of HCV infection on LDs accumulation in cell-culture.

## 1. Introduction

Persistent infection with hepatitis C virus (HCV) is a leading cause of liver fibrosis, cirrhosis, and cancer. Annually, at least 400,000 people die from HCV-related diseases worldwide [1]. Due to the high viral genetic variability, HCV has been classified into eight major genotypes, six of which are considered of epidemiological relevance, and at least 90 subtypes [2,3,4,5]. Strains belonging to different genotypes and subtypes differ by about 30% and 15–20%, respectively, at both the nucleotide and amino acid levels [6]. HCV genotypes 1, 2, and 3 account for more than 80% of global infections. Genotypes and subtypes have been linked to differences in pathogenicity, as well as in treatment response, including the propensity to developing antiviral resistance [6,7].

A hallmark of HCV infection is the presence of steatosis in the liver, which is thought to be linked to liver disease and can be detected in up to 70% of infected individuals [8]. Steatosis is more frequent and severe in genotype 3 infections, and infected liver cells obtained from genotype 3 patients have been shown to contain higher levels of lipids [9,10]. Steatosis in genotype 3 infections is also correlated to viral load and has been shown to be reduced upon viral clearance, whereas it is not or minimally affected by clearance of other genotypes [11,12,13]. Due to this peculiar association between genotype 3 HCV and liver steatosis, the molecular mechanisms of intracellular lipid accumulation have been more intensely studied using this genotype.

Whereas the association between chronic HCV infection and liver steatosis is well documented, the molecular details of HCV-induced intracellular lipid accumulation remain unclear. Several mechanisms have been proposed to explain the increased lipid accumulation in HCV infected patients, including reduced β-oxidation, increased lipid biosynthesis, and interference with VLDL release [10,14]. However, these are mechanisms involved in the metabolic syndrome induced by all HCV genotypes and cannot explain the peculiarities of steatosis described in genotype 3 infections [15]. A direct correlation between the viral load, steatosis levels, and clearance was found in genotype 3 infected patients [15,16].

The expression of HCV Core in cell culture can induce accumulation and redistribution of lipids in lipid droplets (LDs), suggesting a direct steatogenic effect of HCV through the production of viral proteins [17,18]. In particular, the in vitro expression of Core protein from genotype 3a viruses produced higher levels of LDs accumulation in human Huh7 hepatoma cells and larger droplets compared to other genotypes. However, the levels of intracellular lipid accumulation did not correlate with the steatosis levels observed in patients [17,19,20]. A study using a genotype 3a cell-culture adapted virus found the accumulation of higher levels of LDs in Huh7 derived cells, but the comparison was limited to JFH1 (genotype 2a) [21]. Interestingly, a recent study that analyzed liver biopsies from patients with HCV genotype 1 and 3 infections found that the frequency of infected cells with increased LDs accumulation was comparable between the two genotypes and did not correlate with the level of steatosis [22]. In the same study, genotype 3 infected cells contained larger LDs overall, rendering them more easily recognizable as steatotic in histological analyses. Therefore, the study suggests that the molecular mechanisms underlying LDs accumulation are similar in HCV infections with genotype 3a and non-3a but are enhanced in 3a infections due to the presence of larger, more recognizable LDs.

The life cycle of HCV is highly dependent on lipid metabolism, since the virus utilizes cellular lipids for several essential steps, including particle assembly and release [19]. HCV relies on intracellular LDs for accumulation of Core proteins and packaging of viral genomes in the early stages of particle formation [14]. Viral proteins, such as Core and NS5A, have been shown to stimulate the production and/or accumulation of LDs in cell cultures infected with HCV [23]. Moreover, during HCV replication the Core protein is mobilized from the endoplasmic reticulum (ER) to LD upon synthesis for temporary storage, and then transported back to the ER for the assembly of novel viral particles [19,24]. It has been suggested that large amounts of Core on intracellular LDs, as observed during culture infections with the original JFH1 strain, are suggestive of inefficient viral production, whereas the opposite is observed in Jc1 (J6/JFH1 variant of genotype 2a) infections which associate to high levels of particle production [25]. Since Core has also been shown to affect LDs formation and accumulation, this might suggest a link between lipid droplet metabolism and viral production regulation [23].

In this study, we utilized recently developed full-length infectious cell culture systems representing different HCV genotypes and subtypes to investigate LDs accumulation and localization in infected Huh 7.5 cells [26]. Using three-dimensional confocal microscopy, we comparatively analyzed the total and relative lipid volume and its distribution in relation to Core and ER-associated proteins.

## 2. Materials and Methods

### 2.1. Cell Culture and Infections

Huh 7.5 cells were maintained in DMEM supplemented with 10% FBS, Penicillin 100 U/mL, and Streptomycin 100 µg/mL, as previously described [27]. For infections, 4 × 10^5^ cells were plated in 6-well plates and inoculated with cell cultured HCV 24 h post-seeding at the desired multiplicity of infection (MOI). The infection spread was monitored by immunostaining using an anti-HCV-Core primary antibody (C7-50, Abcam, Cambridge, UK) and AlexaFluor488 (Thermo Fisher, Hvidovre, Denmark) secondary antibody with Hoechst counter-staining. Cells were split every 2–3 days and the removed cells were used for flow-cytometry analysis at every time point. For microscopy image analysis, cells were collected 24 h post-infection, plated on 12-chamber slides, and fixed 24 h later.

### 2.2. Flow Cytometry Analysis

Cells were trypsinized and washed in PBS, fixed in 4% formaldehyde for 10 min, and washed again in PBS. Cells were then stained and permeabilized in PBS with 0.5% saponin, 1% BSA, and 0.2% skimmed milk containing mouse-anti-Core antibody (C7-50, Abcam, Cambridge, UK) for 1 h at room temperature. After washing with PBS, cells were incubated with allophycocyanin (APC)-conjugated anti-mouse antibody and Bodipy 493/505 for 1 h in PBS, washed again with PBS, and run on an LSR Fortessa flow cytometer using FACSDiva 8 (Becton Dickinson, Lyngby, Denmark). Data analysis was performed using FlowJo v10 (Becton Dickinson, Lyngby, Denmark).

### 2.3. Microscopy and Image Analysis

Cells plated on chamber slides were fixed in 4% formaldehyde for 10 min and washed in PBS multiple times. Cell were subsequently stained in PBS containing 0.5% saponin, 1% BSA, 0.2% skimmed milk, and primary antibodies overnight at 4 °C. The primary antibodies used were mouse anti-Core (C7-50, Abcam, Cambridge, UK) at 1:500 dilution and rabbit anti-Calnexin, an ER marker (ab22595, Abcam, Cambridge, UK) at 1:500 dilution. After primary incubation, wells were washed in PBS multiple times at room temperature, followed by secondary incubation with PBS for 1–2 h with AlexaFluor555 anti-mouse (1:1000 dilution), AlexaFluor647 anti-rabbit (1:1000 dilution), Bodipy 493/505 (1:500 dilution), and Hoechst 33342 (1:5000 dilution). After the final wash, cells were mounted using Prolong Diamond (Thermo Fisher, Hvidovre, Denmark) and cured for 24 h in the dark.

Imaging was performed on a Leica TCS SP2 confocal system using a 63× NA 1.4 oil immersion objective, 6× electronic magnification, 1024 × 1024 pixel scanning resolution, resulting in a final pixel size of 46 nm. To avoid biasing the cell selection towards brightest cells only, we picked cells for analysis based solely on the presence of a detectable Core signal. Analyses were conducted on at least 15 different image stacks per HCV strain, each including 30–50 images of four channels each. Multichannel image stacks were de-convolved using Huygens Professional (SVI, Hilversum, The Netherlands) and analyzed using Imaris 9 (Andor, Belfast, UK) paired with MATLAB 2018b (MathWorks, Kista, Sweden), using a custom-made program.

The program performs first signal thresholding on all channels for the entire stack, thus removing background noise from the image data. The thresholded data is then used to calculate co-localization of signal intensity between the Core and LD channels, and between Core and ER channels. Co-localization is calculated as the fraction of a signal in one channel that co-localizes with a signal from the other channel. This parameter indicates the spatial overlap between channels but conveys also information on the amount of signal that is co-localized. Although formally similar to the Manders coefficient, this parameter is calculated using a thresholded signal for both channels, whereas Manders is calculated using no thresholding or one-channel thresholding only [28]. The images are then processed to identify three-dimensional surfaces, in order to generate volumetric representations of the signal in each channel. This analysis is performed using Imaris native surface generation functions, with automatic thresholding. The data produced is then utilized to obtain metadata information on each identified volume and to calculate the co-localization of Core/ER and Core/LD volumes. The volumetric co-localization data is similar qualitatively to data obtained by signal analysis, but it does not take into account the signal intensity and results in a more conservative estimate of co-localization. Finally, single channel thresholded images and co-localization channels are exported for publication. Image composition was done in Adobe Illustrator (Adobe, Copenhagen, Denmark).

### 2.4. Graphing and Statistical Analysis

Graphs and statistical analyses were produced using Prism 8 (Graphpad, San Diego, CA, USA) and assembled with Illustrator (Adobe, Copenhagen, Denmark). When comparing average values across all strains we used analysis of variance (ANOVA) methods to evaluate the likelihood that differences in our observation were not due to chance. Specifically, we utilized ordinary one-way ANOVA assuming Gaussian distribution of our values and performed multiple comparison tests against J6/JFH1 to evaluate individual strains, using Dunnett correction. To evaluate whether different variables were correlated, i.e., they showed co-variation, we utilized a linear correlation analysis. Specifically, averages of different observation parameters were compared across HCV strains using Pearson’s correlation assuming Gaussian distribution. The results were evaluated using the Pearson R^2^ parameter and its *p*-value as an indication of co-variation between the analyzed variables and its statistical significance, respectively.

## 3. Results

### 3.1. Different HCV Strains Induce Variable Levels of LDs Accumulation during Early Infection in Infectious Cell-Culture Systems

We investigated the increase in cellular LDs content produced by different HCV strains during the early stages of infection. Human hepatoma Huh 7.5 cells were infected at MOI < 0.1 with cell-culture-adapted full-length HCV recombinants [26] (Table 1). The low MOI was chosen to better observe lipid accumulation over time. Since there were significant fitness differences between the viruses included in this study, we selected specific MOIs that would result in a comparable viral spread at an early time point.

A total of 12 different HCV strains were tested, including three variants of genotype 1a (TN, H77C, and HCV1) [29,30], four of genotype 2a (J6, J6/JFH1, JFH1, and T9) [31,32,33,34], two of genotype 2b (DH8 and J8) [35], one of genotype 2c (S83) [34], and two of genotype 3a (DBN3a and S52) ([36] and unpublished data).

**Figure 1:**
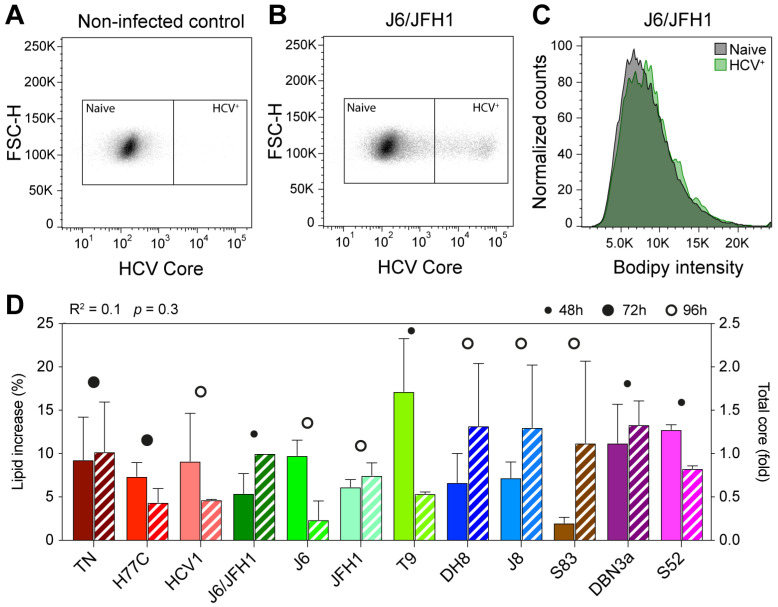
Flow cytometry analysis of early HCV culture infections. Cells infected with different HCV full-length strains were analyzed by flow cytometry at an early infection stage (infection spread ≤ 20%). HCV Core was stained with a mouse anti-Core primary antibody (C7-50, Abcam) and anti-mouse Allophycocyanin (APC)-conjugated secondary antibody; lipid droplets (LDs) were stained using Bodipy 493/505. Graphs show gating of naïve and HCV^+^ cells for non-infected (**A**) and J6/JFH1-infected (**B**) cells selected from one representative experiment out of five independent repeats. The Bodipy median fluorescence intensity (MFI) was then measured on both cell populations (**C**) and used to calculate the increase in LD signal in infected cells. (**D**) Comparison of increase in LD content (solid bars) and relative Core amount (hatched bars) in cells infected with different HCV strains. The Core amount is calculated as normalized Core MFI and depicted here as the fold difference from J6/JFH1 (2a). Red shades represent genotype 1a strains, green 2a, blue 2b, brown 2c, and purple 3a. The data presented in D were used to evaluate the linear correlation between LD increase and Core content, resulting in the r-squared and *p*-value indicated above the graph. Bars represent averages of 2–5 independent measures; error bars represent standard deviation (SD). Circles represent the latest cell harvest time point.

Infected cells were harvested when the HCV infection had spread up to at most 20% of the cell population, which occurred between 24 and 96 h post-infection depending on the infecting strain. After collection, cells were processed for flow cytometry analysis, as described in Materials and Methods. The Bodipy reagent diffuses freely in nonpolar lipids, contained mainly in cytoplasmic LDs, so that the amount of Bodipy retained in fixed cells is proportional to the total amount on neutral lipids present. Thus, by comparing the median fluorescence intensity (MFI) in infected versus non-infected cells within each sample, we could estimate the increase in LDs content following HCV infection (Figure 1A–C).

All 12 strains examined produced an increase in LD content in recently infected cells, however, the extent of the increase was variable (Figure 1D and Table 1). Some viruses, such as S83 (2c) and J6/JFH1 (2a), produced only a small increase in lipid content (2.0 and 5.4%, respectively), while others, such as T9 (2a), led to much higher increases of 17.1%. Genotype 1a viruses led to comparable increases in lipid content: 9.3% for TN, 7.3% for H77C, and 9.1% for HCV1. The two genotype 3a strains included in this study produced increases in LD content of 11.2% (DBN3a) and 12.2% (S52), respectively. These results show that the HCV infection leads to an increase in lipid accumulation in host cells and indicate that the extent of such increase during early infection (24–96 h post-infection) varies among different viral strains, independent of the specific genotype.

The amount of HCV intracellular Core can be used as an indirect marker for viral replication [37]. Viral Core production has been linked to the accumulation of LDs in cells infected with HCV [23]. Thus, we calculated the total amount of Core produced in HCV positive cells by evaluating the Core MFI measured in infected cells normalized to non-infected cells. To exclude the possibility that the observed disparities in LD accumulation were due to differences in viral replication capabilities of the various strains, we then compared the increase in lipid content to the total amount of HCV Core protein in each sample (Figure 1D). Linear regression analysis showed a very low correlation between the two values (R^2^ = 0.1), indicating that the extent of lipid increase is not directly correlated to differences in viral replication levels.

### 3.2. Long-Term Accumulation of Cellular Lipids Does Not Correlate to the Kinetics of Infection Spread

**Figure 2:**
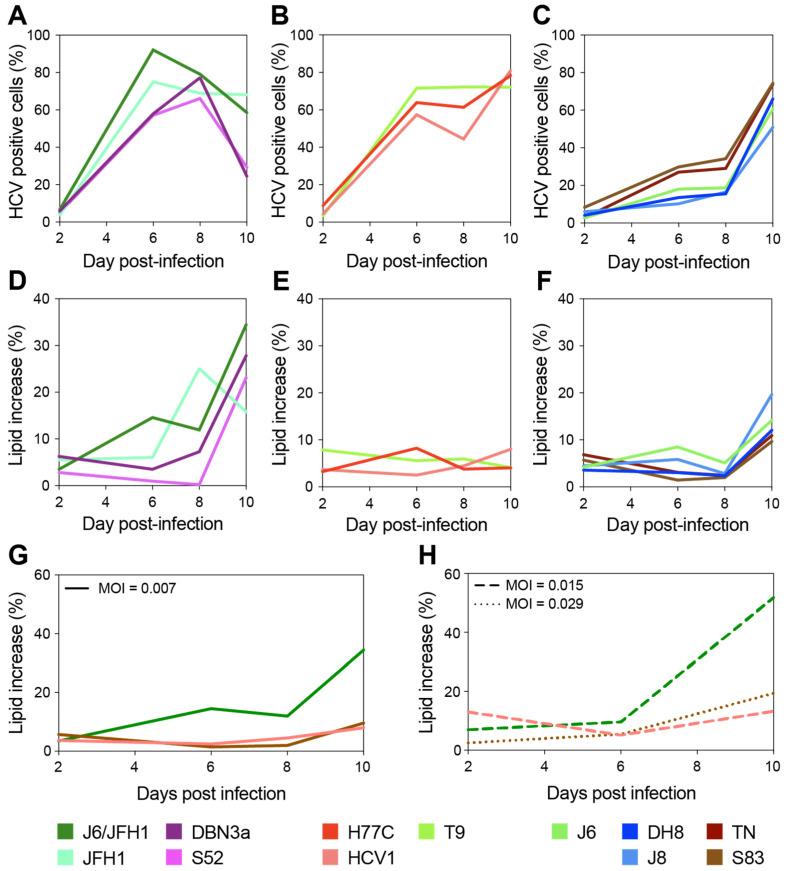
Long-term evaluation of HCV infection spread and LD content increase. Cells infected with different HCV full-length strains were analyzed by flow cytometry every 2–3 days for up to 10 days, and staining was performed as described above (see Figure 1). Cell populations were gated and analyzed as described in Figure 1. A first group of HCV strains showed fast spread (**A**) and high levels of LD increase (>40%) (**D**); a second group also displayed fast spread (**B**) but showed very low accumulation of LD (<10%) (**E**); a third set showed slow spread (**C**) and intermediate levels of LD increase (10 to 20%) (**F**). Three strains, one from each group, were used to assess whether the viral infecting dose had an effect on the observed difference in LD accumulation. Infections with HCV1 (1a), J6/JFH1 (2a), and S83 (2c) were performed at different MOIs (0.007 in panel (**G**); ≥0.015 in panel (**H**)) and monitored for up to 10 days as described above. Colors represent different HCV strains; line styles indicate different MOIs.

To further clarify the relation between the viral strain and intracellular LDs accumulation, we next monitored lipid accumulation during long-term infections (10 days). Cells were infected at low MOI (0.007) and examined every 2–3 days by flow cytometry analyses until the infection had spread to the majority of the culture. The frequency of HCV positive cells and lipid content increase, measured as described above, were evaluated for each time point and sample (Figure 2A–F).

Based on the LD accumulation and infection spread, the viruses could be roughly divided into three groups. A first group showed relatively fast spread kinetics and reached high levels (>40%) of LD accumulation (Figure 2A,D). The second group spread almost as fast as the first group but showed low and constant levels (<10%) of LD accumulation (Figure 2B,E). Finally, a third group displayed slower spread kinetics and reached intermediate levels (10 to 20%) of LD accumulation (Figure 2C,F).

Interestingly, the lipid increase measured at full infection spread did not seem to correlate to the increase detected during early infection (Figure 1 and Table 1). Moreover, as hinted by the experiments shown in Figure 1D these data indicate no general correlation between the spread of viral infection (measured by HCV antigen levels) and LD accumulation.

A direct comparison of the level of LD accumulation between day 2 of the 10-day experiment (Figure 2D–F) and previous analyses of early-stage infections (Figure 1D) is made difficult by the different MOIs used, yet small discrepancies within the same strains could be observed. In particular, several strains including T9 showed lower initial LD accumulation in Figure 2 compared to Figure 1. This is most likely due to the higher MOIs used in the experiment shown in Figure 1 for T9, MOI was 0.03 in Figure 1 and 0.007 in Figure 2, as well as the variability observed across replicates, as shown by the large error bars associated with the Figure 1 data.

Thus, to verify whether the initial MOI could affect the level of LD accumulation and the differences observed between strains, we set up another experiment in which we directly compared the infections performed with three strains (one from each of the above defined kinetics groups: J6/JFH1 (2a), S83 (2c), and HCV1 (1a)) at different MOIs (Figure 2G,H). The results demonstrated that the differences in MOI have no effect on the long-term total accumulation of LD, which remained low for HCV1 (1a) and S83 (2c) and high for J6/JFH1 (2a). However, the initial MOI could affect early LD accumulation levels in infected cells.

### 3.3. HCV Core Polymorphisms Are Not Associated with LD Accumulation

**Figure 3:**
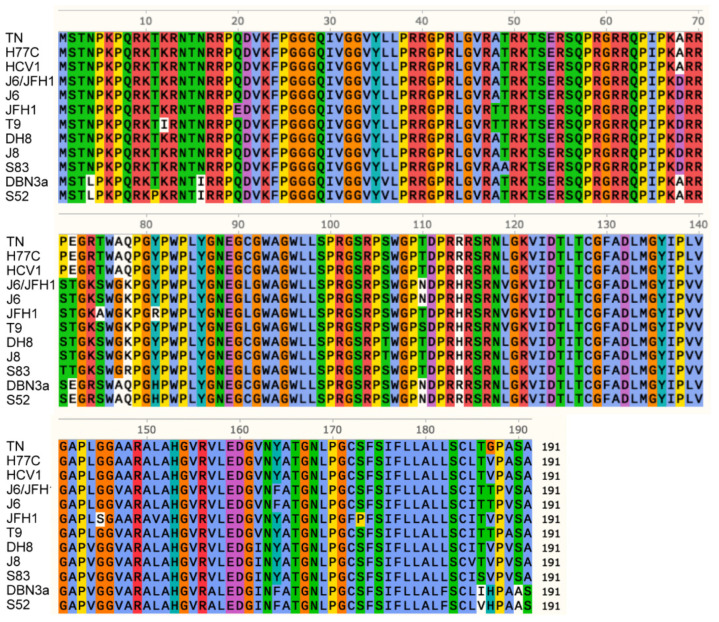
Alignment of amino acid Core sequences from all the analyzed HCV strains. Amino acids are indicated using a single-letter notation. Colors are used to group and distinguish amino acids according to their chemical properties and conservation in the alignment. Different amino acids with the same background color have similar properties; the white background indicates a low conservation. Numbering indicates the amino acid position of Core protein.

The HCV Core expression in cell culture has been shown to produce the accumulation of intracellular LDs [17,18]. Additionally, Core proteins derived from different HCV genotypes have been shown to have differential effects on LD metabolism [17]. To verify whether differences in the Core sequence of the different viruses would recapitulate the observed differences in LD accumulation, we performed an alignment of the amino acid Core sequences of all included HCV strains, obtained by direct sequencing of the cultured viruses (Figure 3).

The analysis revealed a high conservation of the Core region. The majority of polymorphisms detected could be associated with genotype grouping, and there was no clear correlation between specific polymorphisms and a phenotype with high LD accumulation. T9, DBN3a, and S52, for example, had shown the highest LD accumulation in recently infected cells (Figure 1D) but showed no common polymorphisms. In particular, Core domain 2 (aa 118–177), which is responsible for Core association with LDs and ER during viral assembly, showed a high conservation [38]. These findings suggest that LD accumulation in infected cells is not related to specific sequence polymorphisms in the Core protein.

### 3.4. Infection by Different HCV Strains Results in Variable Levels of LDs Accumulation in Cell Culture

**Figure 4:**
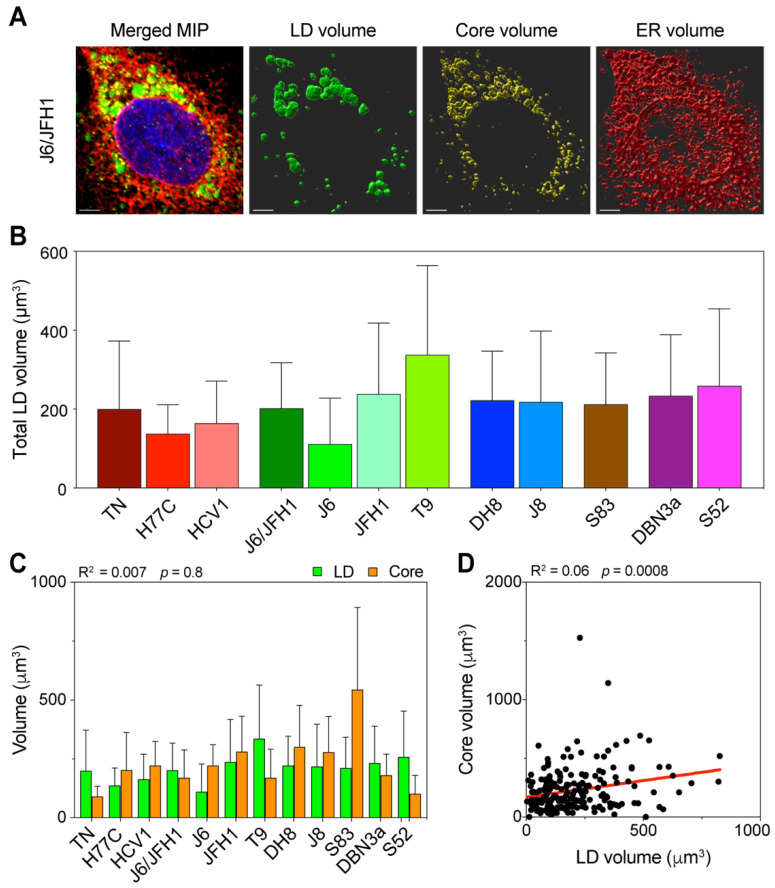
Three-dimensional volume reconstruction and analysis of infected cells. Cells infected with different HCV full-length strains were fixed, stained, and imaged by fluorescence confocal microscopy 48 h after infection. To obtain three-dimensional information of the cellular content, individual cells were imaged in stacks of around 40 planes in four fluorescence channels. HCV Core was stained using C7-50 and AlexaFluor555 antibodies, LDs were stained using Bodipy 493/505, ER was stained using anti-calnexin ab22595 and AlexaFluor647 antibodies, and nuclei were stained using Hoechst. (**A**) Signal intensity data were then used to reconstruct the volumetric distribution of LD, Core, and ER proteins in Imaris using a custom-made script run from MATLAB. The merged picture represents a maximum intensity projection (MIP) of the original stack, illustrating the cumulative signal in all channels. The volume pictures show three-dimensional representations of the signal distribution in the three analyzed channels. The scale bar represents 5 µm. (**B**) Average of total LD volume in cells infected with each HCV strain. Bars represent the mean of at least 15 cells. Error bars represent SD. Color-coding is maintained from Figure 1. (**C**) Comparison of total volumes of LD and Core in the different infections. The data presented were also used to evaluate the linear correlation between LD and Core volumes, resulting in the r-squared and *p*-value indicated above the graph. Bars represent averages of ≥15 cells; error bars represent SD. Colors of Core and LD are unrelated to the HCV strain-associated color, instead they are meant to resemble the fluorochromes used for detection. (**D**) Linear regression analysis of total LD and Core volume in infected cells, irrespective of the infecting HCV strain. Each dot represents one cell (n = 192). The obtained r-squared and *p*-value are indicated above the graph. The regression line is presented in red.

To further investigate the nature and extent of LD formation and accumulation in infectious cell-culture systems, we next analyzed LD morphology in infected cells using high-resolution 3D confocal microscopy. Cells infected with the different HCV strains at MOI < 0.1 were fixed and stained 48 h post-infection and imaged. Image stacks were used to reconstruct the cellular three-dimensional morphology and compute volume occupation of LDs, ER, and Core (Figure 4A).

The distribution of total LD volume average showed significant differences among HCV strains (*p* = 0.02, ANOVA), with an overall trend comparable to the LD increase observed during early infection (Figure 4B). The T9 (2a) strain showed the highest amount of LD, whereas J6 (2a) had the lowest. J6/JFH1 (2a) and JFH1 (2a) together with strains of genotypes 2b and 2c had intermediate amounts, such as the 3a strains. The 1a strains showed the lowest LD amounts overall.

Then, we compared the average total amounts of LD and Core, to determine the correlation between the LD content and viral replication (Figure 4C). The analysis confirmed that the HCV Core production was not directly correlated to the LD intracellular content (R^2^ = 0.007). Furthermore, the lack of correlation between the Core and LD content was even more significant when we compared across all samples, irrespective of the infecting strain (Figure 4D), suggesting that in early infection the amount of intracellular LD is unrelated to the virus replication.

Overall, these results show that different HCV viral strains affect the total LD content of recently infected cells (48 h post-infection) in very distinct ways, irrespective of their replication capacity in the cell-culture. Moreover, this effect was comparable to what was observed in flow cytometry analyses of early infections (24–96 h post-infection).

### 3.5. HCV Induces LD Accumulation by Increasing the Number of Intracellular LDs

**Figure 5:**
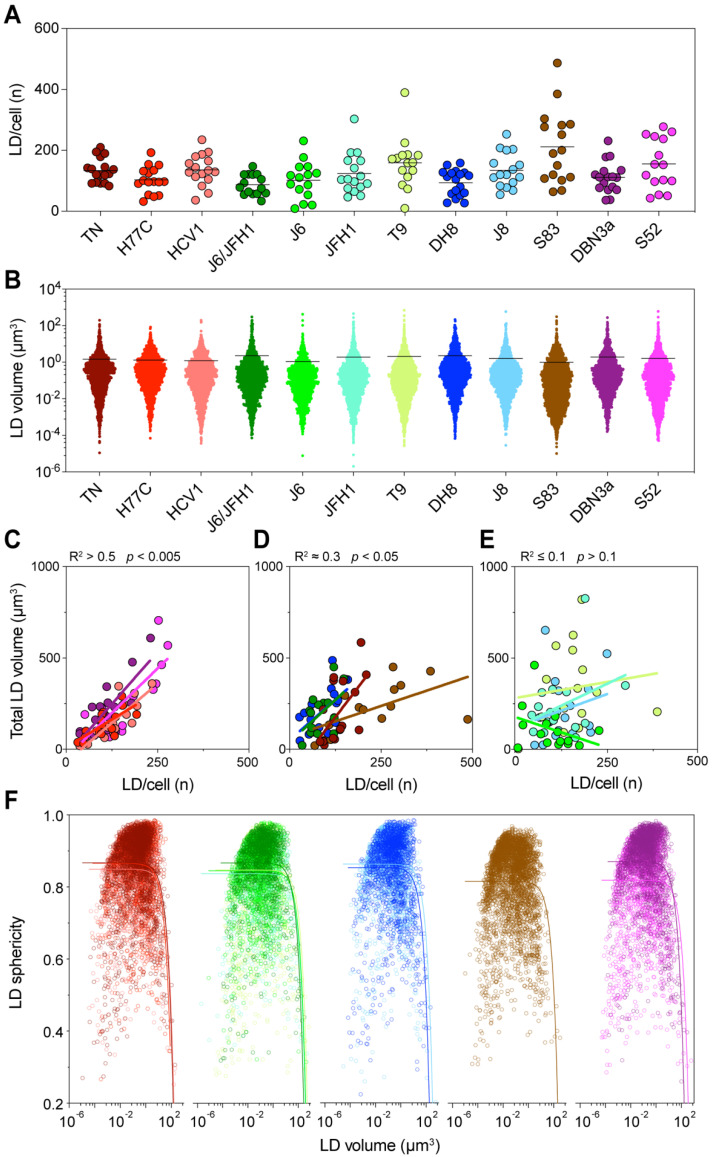
Detailed analysis of intracellular LDs. Lipid droplet statistics obtained through the three-dimensional (3D) image reconstruction described in Figure 4, including the size, number, and shape, were used to investigate the correlation with each other and with different cellular characteristics. (**A**) The graph presents the total number of LDs identified in each cell, grouped according to the infecting HCV strain. Dots represent individual cells; bars indicate the average number of LDs for each infecting strain. (**B**) Distribution of individual LD volumes obtained from all cells infected with each HCV strain. Dots represent individual LDs; bars indicate the average LD volume for each infecting strain. (**C**–**E**) Linear regression analysis of total LD volume against the number of LDs per cell was performed for each infection. The graphs present strains showing high (**C**), low (**D**) or no (**E**) correlation between these values. The obtained r-squared and *p*-value are indicated above the graphs. (**F**) Correlation analysis of LD shape, quantified as sphericity, and LD volume. The graph presents all LDs identified in cells infected with each HCV strain; for readability the data is divided according to the HCV subtype: Red shades represent genotype 1a strains, green genotype 2a, blue 2b, brown 2c, and purple 3a. The colored curves represent regression lines between the LD shape and size. LD volume is presented using a log_10_ scale.

In order to further characterize the LD accumulation in infected cells, we performed single-cell volumetric and morphological analysis of LDs. The volumetric reconstruction permitted by 3D microscopy also allowed the identification and analysis of individual LDs within the infected cells. To clarify the mechanisms of LD accumulation caused by the HCV infection, we first evaluated the total number of LDs within cells infected by different viral strains. The average number of LD per cell showed variability among HCV strains, but could not entirely recapitulate the distribution of total LD volume observed (Figure 5A). In fact, while differences in LD numbers among strains were highly significant (*p* < 0.0001, ANOVA), the strain with the highest mean number of LDs was S83 (2c), which had only showed intermediate levels of total LD volume and a very small increase of LD content in the flow cytometry analyses.

Then, we analyzed the size distribution of individual LD in all cells infected with the same HCV strain (Figure 5B). Similarly, the distribution of LD sizes showed significant differences among HCV strains (*p* = 0.002, ANOVA). However, it could not directly explain the levels of total LD volume observed earlier. Interestingly, in this analysis J6/JFH1 (2a) and DH8 (2b) had the highest average LD sizes, whereas S83 (2c) had the lowest. Thus, an increase in LD numbers or LD individual size could not directly account for the differences in total LD volume observed in cells infected with the different HCV strains. However, when we compared the number of LDs to their total volume, we could detect distinct patterns in different strains (Figure 5C–E). A first group of strains showed a very good correlation between the LD number and volume (R^2^ > 0.5), suggesting that in these cases the increase in total LD volume was caused by an increase in the LD number. A second group showed a low but significant correlation (R^2^ ≈ 0.3), indicating that the increase in LD number could only partially explain the increase in total volume. Finally, a third group displayed no correlation between the two values (R^2^ ≤ 0.1). Interestingly, the strains with the lower correlation values seemed to have either very high numbers or a very high total volume of LD.

Thus, we analyzed the correlation between the individual LD size and shape, evaluated as the sphericity of the LD (Figure 5F). The analysis showed that most LDs were spherical or almost spherical (sphericity = 1) across a wide range of sizes, implying that they were individual LDs. However, the sphericity parameter dropped quickly once the LD size reaches extreme values, suggesting that large LDs are in fact aggregates of multiple fused LDs, which are thus mis-counted as individual bodies (Figure 5F).

Taken together, these data indicate that the increase in LD amount in infected cells is due to an increase in the number of individual LDs, irrespective of the infecting strain. Distinct HCV strains will induce higher or lower LD numbers. However, when the number of LDs reaches the highest levels this will lead to their fusion resulting in apparent lower LD counts.

### 3.6. Intracellular Core Distribution Is Independent of LD Content

**Figure 6:**
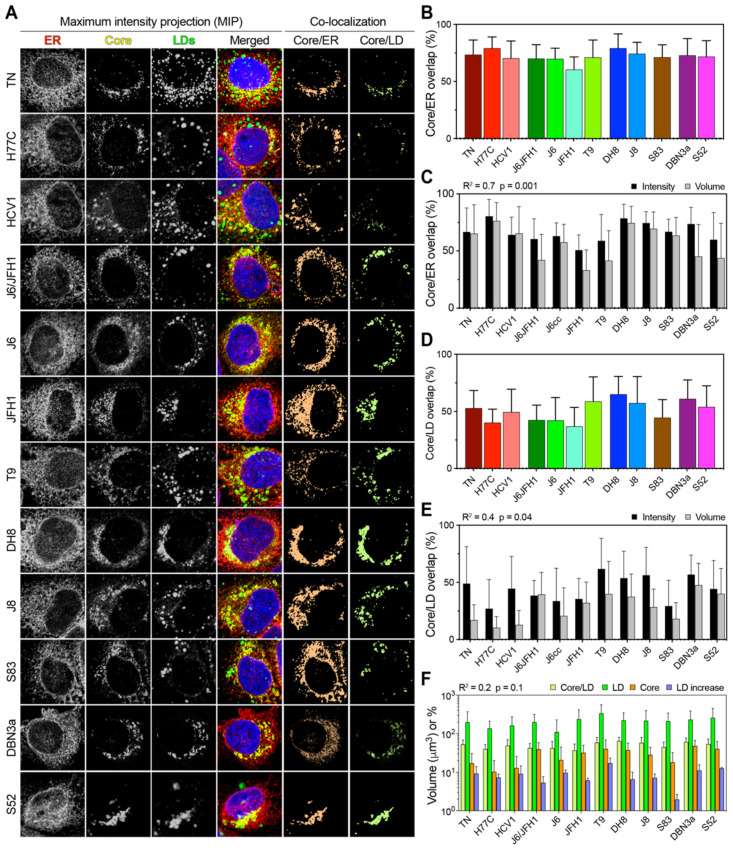
Co-localization analysis of Core, LD, and ER in HCV infected cells. (**A**) Representative images of Core, LD, and ER channels, together with a merged overlay of all channels including the nucleus, are presented as the MIP for each HCV infecting strain. The signal intensity from the first three channels was used to calculate the co-localization of Core/LD and Core/ER as the amount of signal overlap between their channels. For readability, the co-localization channels are depicted here as flattened color channels, without intensity variation. (**B**) Co-localization of the Core and ER presented as the fraction of total Core signal that overlap with any ER signal. Bars represent averages of ≥15 cells; error bars represent SD. (**C**) Correlation analysis of Core/ER co-localization performed using either intensity or volumetric data. Bars represent averages of ≥15 cells; error bars represent SD. The obtained r-squared and *p*-value are indicated above the graph. (**D**) Co-localization of Core and LD presented as the fraction of total Core signal that overlap with any LD signal. Bars represent averages of ≥15 cells; error bars represent SD. (**E**) Correlation analysis of Core/LD co-localization performed using either intensity or volumetric data. Bars represent averages of ≥15 cells; error bars represent SD. The obtained r-squared and *p*-value are indicated above the graph. (**F**) Correlation analysis between Core/LD intensity co-localization values and total LD volume, total Core volume, or LD increase, as depicted in Figure 1 and Figure 4. Volumes (LD and Core) and percentages (Core/LD and LD increase) are all presented using the same log_10_ scale for ease of comparison. Bars represent averages of ≥15 cells; error bars represent SD. All three linear regression analyses gave similar r-squared and *p*-values, as indicated above the graph.

The expression of HCV Core of different genotypes has been shown to affect the amount and size on intracellular LD [17]. In addition, the HCV Core interacts directly with LD during viral morphogenesis and possibly participating in LDs regulation. To investigate whether the Core and LDs interacted differently in different HCV genotypes, we evaluated the correlation between LD accumulation and Core distribution in infected cells, using three-dimensional image reconstruction and co-localization analyses (Figure 6A). First, we evaluated the fraction of Core localized on the ER by calculating the intensity overlap of the Core and calnexin (ER-associated protein) antibody signals (Figure 6B).

This analysis takes into account both positional overlap and absolute intensity, thus providing an accurate estimate of the amount of Core located on or in proximity of the ER. All HCV strains showed extensive Core localization on the ER, with only JFH1 displaying significantly lower ER localization in reference to the other strains (*p* < 0.01; ANOVA). Then, we performed a volumetric co-localization analysis of Core and ER, calculating the fraction of Core volume that co-localizes with the ER volume. This analysis ignores the actual intensity of the signal, but only evaluates whether a signal from one channel that is above the threshold co-localizes with the signal from the second channel, thus providing a more conservative estimate of signal overlap. Although the volumetric method resulted in lower values overall, the two approaches yielded comparable results (R^2^ = 0.7), supporting the finding that Core is mostly localized on the ER in all HCV infections (Figure 6C).

Next, we analyzed the distribution of Core on LDs using the intensity overlap and found significant differences among HCV strains (*p* < 0.0001; ANOVA) (Figure 6D). Strains of genotype 3a, 2b, and the genotype 2a T9 strain showed the highest co-localization with LDs. A comparison with volumetric co-localization values showed a moderate and significant correlation (R^2^ = 0.4), confirming that Core localizes on LDs to different extents in different HCV strains during infection in cell-culture (Figure 6E).

To evaluate whether the Core distribution on LDs is directly associated with the total Core or LD content, we performed the linear regression analysis of Core/LD co-localization against the LD increase, total LD volume, and total Core volume (Figure 6F). All three analyses yielded similar R^2^ values of about 0.2, indicating a poor correlation between the analyzed parameters. Our results from the imaging analysis indicate that the HCV Core localized on LDs to a variable extent in infections with different strains. However, the extent of LD/Core co-localization was not correlated with either the LD increase or total content, nor with the total Core amount.

## 4. Discussion

Steatosis of the liver has long been recognized as a hallmark of HCV infection, especially in genotype 3a infections. The molecular mechanisms underlying the development of liver steatosis in HCV infection, however, remain unclear. One working hypothesis is that viral-induced intracellular lipid accumulation would result in a steatotic liver, with genotype 3a infections thus producing higher levels of intracellular lipid accumulation than other genotypes [9]. This working model is supported by early studies showing that the HCV Core expression leads to an increase in intracellular LDs accumulation [39]. When the Core from different patient strains were expressed in cell culture, the genotype 3a Core expression resulted in higher levels of lipid accumulation in cells compared to other genotypes [17,40]. However, the molecular details underlying such differences have proven difficult to evaluate in cell culture, partly due to a lack of comparative studies examining multiple genotypes simultaneously, and for the lack of systems recapitulating the complete life cycle of multiple genotypes in culture. Primary human hepatocytes (PHHs) would represent a more physiological culture system to compare LD accumulation in infected cells to observations from clinical samples. However, PHHs typically show reduced susceptibility to infection by cell-culture adapted HCV strains [41]. Therefore, for this project, we elected to utilize the gold-standard Huh 7.5 cell line, which has been proven as a reliable model for the study of LD metabolism in HCV infection [42,43,44,45].

In this study, we set out to investigate and characterize lipid accumulation during infection with cell-culture adapted HCV strains of several genotypes and subtypes, which together account for the majority of human infections worldwide. We found differences in lipid accumulation among the different HCV strains. By measuring lipid accumulation by both flow cytometry and 3D microscopy, we found a broad variation across HCV strains and found that strains of genotype 3a did not lead to uniquely high levels of intracellular LDs among genotypes. The differences in our findings compared to previous reports are most likely due to the cell-culture system used in our study, which allows observation of the full viral life cycle, and the different 3a strains evaluated here. The expression of not only the Core, but also nonstructural proteins involved in virion assembly, is arguably responsible for the modulation of the effect of Core on the LDs metabolism.

Interestingly, genotype 3a strains did produce higher levels of lipid accumulation in long-term culture assays, suggesting that longer culture times might better recapitulate the effects observed with Core-only expression systems. Additionally, our data showed that inter-strain differences persisted irrespective of the amount of viral inoculum. This further supports the idea that the levels of LD accumulation during HCV infection are determined not only by the apparent replication efficacy of the viral strain, but rather is a complex process involving how the specific strain might interact with diverse host-factors, differences that could be driven by the genetic diversity of HCV. The increased sequence variability that we observed among Core proteins in domain 1, which has been proposed to interact with several host-cell factors, lend support to this hypothesis [46].

A cell-culture study comparing genotypes 3a and 2a (strains S310 and JFH1) reported higher levels of LD accumulation in cells infected with the 3a virus [21]. In addition to studying only two strains, the study relied on a two-dimensional image analysis to assess the LD accumulation. The difference in the analysis method and the strain used might explain the discrepancy with our results, as we compared data across several strains and used volumetric data from whole cell analysis. Our automated three-dimensional image analysis provided unbiased counts and LD volumes within entire cells, resulting in a more extensive representation of cellular behavior.

Our results, on the other hand, are consistent with a growing number of studies suggesting that while HCV genotype 3a could lead to high levels of lipid accumulation overall, its effect cannot be explained solely by the Core sequence [17,22,43,47]. Indeed, we observed extreme low variability in Core domain 2 among our HCV strains, indicating that the Core interaction with LDs and ER is most likely not directly modulated by the protein sequence. Most importantly, histological steatosis is not recapitulated by the Core sequence alone, supporting the idea that intracellular lipid accumulation and liver steatosis are modulated by a broader network of protein-protein interactions involving but not restricted to the HCV Core [22,47].

Most previous studies, both in vitro and in vivo, agree that the genotype 3 infection leads to a higher accumulation of LDs with increased size and thus to an overall higher LD intracellular content [9,17,22,40]. In our study, this unique phenotype of genotype 3 was not apparent, since the distribution of individual LD sizes was comparable across strains and genotypes. On the contrary, the genotype 3a strains were among the few showing a significant linear correlation between the total LD content and number of intracellular LDs, indicative of an increase in the LD number rather than size. Our analyses also highlighted possible technical issues related to the detection of large LDs by microscopy, indicating that multiple small LDs can be erroneously identified by image analyses as single large droplets thus leading to miscalculations. These findings suggest that an overall increase in the number of LDs is responsible for the increase in lipid content irrespective of the infecting genotype.

A recent study comparing liver biopsies from HCV patients infected with genotype 1 or 3 found that cells infected with genotype 3 had larger LDs than cells from genotype 1 patients. However, the irregular shape of LDs in genotype 3 samples could indicate that those were in fact derived from larger numbers of smaller LDs [22]. In this regard, it is interesting that another study using transmission electron microscopy (TEM) to measure the intracellular LD content did not find significant differences between genotype 3a and non-3a, possibly since TEM allows a more precise identification of individual LDs [47]. These results also underscore that care should be taken when interpreting three-dimensional data using a bi-dimensional representation and that the volumetric analysis seems better suited for cellular content determination.

During the HCV replication cycle, the Core protein shuttles from the site of production on the ER to the LD surface for temporary storage, and then back to the ER for virion assembly [48]. The expression of HCV Core alone leads to the accumulation of intracellular LDs, as previously mentioned, but also invariantly leads to the accumulation of Core uniquely on the surface of the LDs themselves [9,17,39,40]. On the other hand, the expression of full-length HCV in cell culture leads to only a partial accumulation of Core on LDs [49]. It is generally assumed that higher levels of LD-associated Core are indicative of reduced viral production, as observed in the original JFH1 infections [25]. The analysis of Core distribution in genotype 3a (strain S310) infections in culture surprisingly showed almost no LD localization [21]. The analysis of all our strains, including two other genotype 3a strains, indicated that the Core was mostly localized on the ER, and to different extents on LDs. Our JFH1 strain was adapted to the cell culture, which might explain the higher level of ER localization and lower levels of Core localization we measured compared to other studies that used the original non-adapted clone [21,25]. Our data thus indicate that our full-length cell-culture systems allow for the effect of Core on lipid accumulation and viral production to be modulated by sufficient interactions with other viral components and host factors, making them valuable tools to further investigate the interaction between the HCV infection and cellular lipid metabolism.

One enticing hypothesis regarding the induction of LD accumulation by HCV is that the increase in total LD surface would in turn support increased viral production by facilitating a novel virion assembly at multiple sites. Although few strains seem to support this hypothesis (T9 (2a), DBN3a (3a), S52 (3a)), we could not detect any relevant correlation between the Core/LD co-localization and either total LD content or LD increase, or even total Core levels. Our analyses also showed that the lipid accumulation on LDs was not associated to the levels of Core expression, as had been previously reported [39]. These results promote the idea that LD accumulation is induced by HCV through mechanisms which are not correlated to viral replication efficiency only. In this regard, recent results indicating that various Core domains can independently affect LD induction and Core/LD localization seem to support our findings linking sequence diversity to differential LD production and Core localization as the driver force behind the differences observed in the strains used in this study [43,44,50]. In addition, our system seems to better recapitulate observations obtained from clinical studies regarding lipid accumulation, compared to HCV Core expression systems, thus representing a good candidate to further investigate the molecular mechanisms of HCV lipid accumulation.

We found that different HCV strains induce the accumulation of LDs to distinct levels in infected cells, independently of viral replication levels. However, LD accumulation is not dictated simply by the HCV genotype as commonly assumed, which is of particular relevance for genotype 3a. This observation might apply to liver steatosis, as well. More complex interactions than the Core-LD association are responsible for lipid accumulation, possibly involving host-specific factors. The accumulation of LDs is achieved mainly through an increase in the number of LDs, which can coalesce into larger droplets. The increased overall LD surface does not correlate with higher levels of Core/LD co-localization, indicating that the LD accumulation has limited direct linkage to viral replication levels. These findings prompt further investigation into inter-molecular interactions, possibly between the domain 1 of Core and cellular host factors, or between different HCV proteins, to further unravel the viral mechanisms involved in cellular lipid dysregulation.

## Figures and Tables

**Table 1:** Cumulative results of flow and microscopy analyses for all studied hepatitis C virus (HCV) strains.

HCV Strain	HCV Genotype	MOI	LD Increase ^a^	LD Volume ^b^	LD Number	LD Size ^b^	Core Volume ^b^	Core on ER ^a^	Core on LD ^a^
TN	1a	0.030	9.3 (4.9)	200 (174)	134 (40)	1.5 (7.0)	91 (43)	74 (13)	53 (15)
H77C	1a	0.033	7.3 (1.6)	137 (75)	104 (44)	1.3 (4.1)	203 (160)	79 (10)	40 (12)
HCV1	1a	0.015	9.1 (5.6)	164 (107)	136 (51)	1.2 (7.0)	222 (103)	70 (15)	50 (20)
J6/JFH1	2a	0.015	5.4 (2.3)	201 (116)	88 (34)	2.3 (14.4)	170 (119)	70 (12)	42 (13)
J6	2a	0.015	9.8 (1.8)	111 (117)	102 (61)	1.1 (12.5)	222 (90)	70 (9)	42 (20)
JFH1	2a	0.091	6.1 (0.9)	237 (181)	125 (67)	1.9 (15.0)	281 (150)	61 (11)	37 (17)
T9	2a	0.030	17.1 (6.1)	337 (227)	159 (84)	2.1 (21.7)	170 (122)	71 (15)	59 (21)
DH8	2b	0.028	6.6 (3.4)	222 (126)	94 (42)	2.3 (10.3)	301 (178)	79 (13)	65 (16)
J8	2b	0.021	7.2 (1.8)	218 (180)	135 (57)	1.6 (14.4)	279 (152)	74 (10)	58 (23)
S83	2c	0.029	2.0 (0.7)	212 (131)	212 (121)	1.0 (7.7)	545 (349)	71 (11)	45 (16)
DBN3a	3a	0.080	11.2 (4.5)	233 (156)	111 (51)	1.9 (10.3)	181 (90)	73 (15)	61 (16)
S52	3a	0.030	12.8 (0.6)	2596 (196)	156 (83)	1.7 (15.5)	102 (77)	72 (14)	54 (19)
ANOVA	*p*-value		0.0365	0.0169	<0.0001	0.0018	<0.0001	0.0087	<0.0001

Values in parenthesis represent standard deviation; ^a^ = %; ^b^ = µm^3^.

## Data Availability

The MATLAB/Imaris script used for the analysis is available on Github via Zenodo. Available online from 1 December 2020: https://doi.org/10.5281/zenodo.3932312. The microscopy data analyzed and presented in this study are available on request from the corresponding author. The data are not publicly available due to size restrictions.

## Non-Standard Abbreviations

Hepatitis C virus (HCV); lipid droplets (LDs); endoplasmic reticulum (ER); allophycocyanin (APC); median fluorescence intensity (MFI); standard deviation (SD); maximum intensity projection (MIP).
